# Using Methacryl-Polyhedral Oligomeric Silsesquioxane as the Thermal Stabilizer and Plasticizer in Poly(vinyl chloride) Nanocomposites

**DOI:** 10.3390/polym11101711

**Published:** 2019-10-18

**Authors:** Yu-Kai Wang, Fang-Chang Tsai, Chao-Chen Ma, Min-Ling Wang, Shiao-Wei Kuo

**Affiliations:** 1 Department of Materials and Optoelectronic Science, Center of Crystal Research, National Sun Yat-Sen University, Kaohsiung 80424, Taiwan; gba01123@gmail.com; 2 Hubei Key Laboratory of Polymer Materials, Key Laboratory for the Green Preparation and Application of Functional Materials (Ministry of Education), Hubei Collaborative Innovation Center for Advanced Organic Chemical Materials, School of Materials Science and Engineering, Hubei University, Wuhan 430062, China; 3 UPC Technology Corporation, No.3, Kung-Yeh 2nd Rd., Linyuan Dist., Kaohsiung 832, Taiwan; cj.maa@upc.com.tw (C.-C.M.); vini.wang@upc.com.tw (M.-L.W.); 4 Department of Medicinal and Applied Chemistry, Kaohsiung Medical University, Kaohsiung 807, Taiwan

**Keywords:** POSS, poly(vinyl chloride), plasticizer, nanocomposites

## Abstract

In this study, we investigated the influence of methacryl-functionalized polyhedral oligomeric silsesquioxane (MA-POSS) nanoparticles as a plasticizer and thermal stabilizer for a poly(vinyl chloride) (PVC) homopolymer and for a poly(vinyl chloride)/dissononyl cyclohexane-1,2-dicarboxylate (PVC/DINCH) binary blend system. The PVC and the PVC/DINCH blend both became flexible, with decreases in their glass transition temperatures and increases in their thermal decomposition temperatures, upon an increase in MA-POSS content, the result of hydrogen bonding between the C=O groups of MA-POSS and the H–CCl units of the PVC, as determined using infrared spectroscopy. Furthermore, the first thermal decomposition temperature of the pure PVC, due to the emission of HCl, increased from 290 to 306 °C, that is, the MA-POSS nanoparticles had a retarding effect on the decomposition of the PVC matrix. In tensile tests, all the PVC/DINCH/MA-POSS ternary blends were transparent and displayed flexibility, but their modulus and tensile strength both decreased, while their elongation properties increased, upon an increase in MA-POSS concentration, both before and after thermal annealing. In contrast, the elongation decreased, but the modulus and tensile strength increased, after thermal annealing at 100 °C for 7 days.

## 1. Introduction

Poly(vinyl chloride) (PVC) is one of the most commonly employed polymeric materials. It is used widely in packing materials, toys, healthcare, electric insulation, automobiles, and interior decorations, because of its high transparency, inertness, and reasonable mechanical properties [1,2,3,4,5]. Nonetheless, the intrinsic rigidity of PVC limits its end-user applications, because of its high glass transition temperature (*T*_g_, ca. 89 °C). Therefore, PVC is often blended commercially with various plasticizers, e.g., di-(2-ethylhexyl)phthalate (DEHP) [1]. A serious shortcoming of DEHP, however, is its leachability from PVC materials upon contact with tissues or body fluids [6,7].

Dissononyl cyclohexane-1,2-dicarboxylate (DINCH) is a promising substitute plasticizer for DEHP in PVC; it exhibits high biodegradability and low environmental persistence compared with DEHP, the result of replacing the benzene ring in DEHP with a cyclohexane ring in DINCH [1]. Nevertheless, this substitution can result in PVC/DINCH blend systems possessing thermal stability lower than that of corresponding PVC/DEHP blends. As a result, various nanofillers have been tested as plasticizers to enhance the thermal stability of PVC [8,9,10]. Ideally, these nanofillers would function as plasticizers, while inhibiting the degradation of the polymer and the release of HCl.

Polyhedral oligomeric silsesquioxane (POSS) derivatives are organic/inorganic hybrid materials that have been widely dispersed in polymeric matrices by taking advantage of both chemical and physical bonding interactions [11,12,13,14,15,16,17,18,19,20,21,22,23,24,25,26]. Such POSS-related polymer nanocomposites can exhibit enhanced strength, rigidity, decomposition temperature, and modulus, and decreased viscosity, flammability, and surface free energy, depending on the degree of dispersion of POSS nanoparticles in the polymeric matrix [27,28,29,30,31]. In the case of physical bonding, the POSS units can be dispersed through solvent-casting or melting/mixing blending approaches (i.e., without covalent bonding). Blending with POSS as a plasticizer can increase thermal decomposition temperature, enhance impact strength, and lower the value of *T*_g_ of PVC [32,33,34,35]. Various functional groups (e.g., methacryl and ethylene glycol units) have been appended to POSS nanoparticles for incorporation into PVC matrices. Because the thermal decomposition temperature with 10 wt% loss of the ethylene glycol unit (*T*_d10_ = 250 °C) [35] is lower than that of the methacryl unit (*T*_d10_ = 420 °C) [33,34], in this study, we chose to combine methacryl-POSS (MA-POSS) nanoparticles with DINCH to use as plasticizers for a PVC matrix. The incorporation of MA-POSS in PVC reduces both the primary and secondary transition temperature. In addition, the ternary blend of PVC/MA-POSS/DOP could reduce *T*_g_ behavior near room temperature with desirable ductile behavior. To the best of our knowledge, this paper is the first to describe the incorporation of combinations of DINCH and MA-POSS within PVC matrices. We have employed differential scanning calorimetry (DSC), dynamic mechanical analysis (DMA), Fourier-transform infrared (FTIR) spectroscopy, thermogravimetric analysis (TGA), and tensile tests to characterize the glass transition and thermal decomposition temperatures, hydrogen bonding interactions, and mechanical properties of binary blends of PVC/MA-POSS and ternary blends of PVC/DINCH/MA-POSS.

## 2. Materials and Methods

### 2.1. Materials

**Scheme 1:**
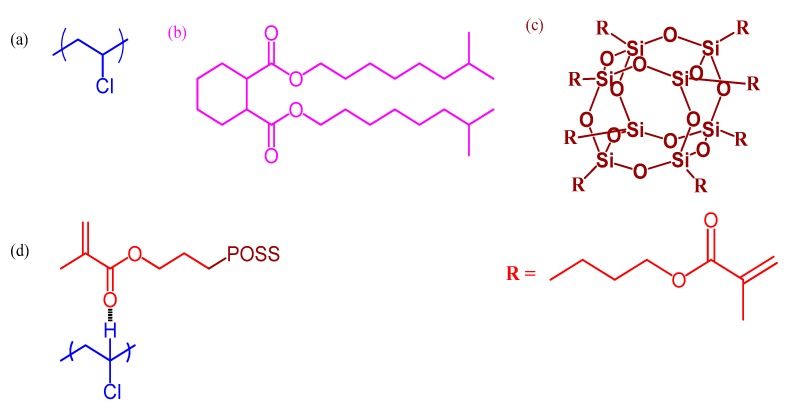
(**a**–**c**) Chemical structures of (**a**) Poly(vinyl chloride) (PVC), (**b**) Dissononyl cyclohexane-1,2-dicarboxylate (DINCH), and (**c**) methacryl-polyhedral oligomeric silsesquioxane (MA-POSS). (**d**) Hydrogen bonding interaction in PVC/MA-POSS blends.

PVC (Scheme 1a) and tetrahydrofuran (THF) were purchased from Sigma–Aldrich. DINCH (UniHydro UN899, Scheme 1b)) was supported by UPC Technology Corporation. The average molecular weight of the PVC used in this study was approximately 90,000, with a polydispersity index (PDI) of 2.25 by Sigma–Aldrich. MA-POSS (Scheme 1c) was purchased from Hybrid Plastics; this compound did not have a crystalline structure, but featured a glass transition temperature of −55 °C (Figure S1) and was a pale-yellow heavy oil [33,34].

### 2.2. PVC/DINCH, PVC/MA-POSS, and PVC/DINCH/MA-POSS Blend Films

Binary blends of PVC/DINCH and PVC/MA-POSS and ternary blends of PVC/DINCH/MAPOSS were prepared through solvent-blending, using THF as the solvent. A solution containing 10 wt% of the polymer mixture was stirred for 24 h and then the solution was poured into a Teflon mold. The solvent (THF) was evaporated slowly over 3 days at room temperature. The resulting blend film was heated under a vacuum at 50 °C for 2 days and then at 80 °C for 2 days to ensure the total removal of any residual solvent. Samples for DSC, TGA, DMA and tensile testing were pelletized, compression-molded into disks (160 °C, 3 min), and then machined into suitable specimens for testing.

### 2.3. Characterization

The glass transition temperatures of all the blend systems were measured using DSC and DMA. DSC was performed using a TA-Q20 instrument, operated at a heating rate of 20 °C/min between 25 and 150 °C under a N_2_ atmosphere, and the sample was quickly cooled to −90 °C after the first scan. The *T*_g_ values were determined at the midpoint of the transition temperature, with a scan rate of 20 °C/min, in the range −90–200 °C. The dynamic mechanical properties were characterized using a DuPont 2980 dynamic mechanical analyzer, with the blend sample mounted on the single cantilever clamp. A constant strain amplitude of 2.0% and a frequency of 1 Hz were applied, with a heating rate of 2 °C/min between −60 and +120 °C. The thermal stabilities of the blend samples were determined using a TA Q-50 thermogravimetric analyzer, operating within the temperature range 30–800 °C at a heating rate of 20 °C/min, under N_2_. FTIR spectra samples of the blend were recorded using a Bruker Tensor 27 spectrophotometer. A total of 32 scans were collected at a spectral resolution of 4 cm^−1^. The FTIR samples were measured using conventional KBr disk method, similarly to the preparation of the bulk sample. The film used in this work was sufficiently thin to obey the Beer–Lambert law. Tensile tests were performed using an Instron machine. Five different blend samples of each composition were examined at a tensile rate of 1 cm/s.

## 3. Results

### 3.1. Thermal Analysis of PVC/DINCH Blends

**Figure 1:**
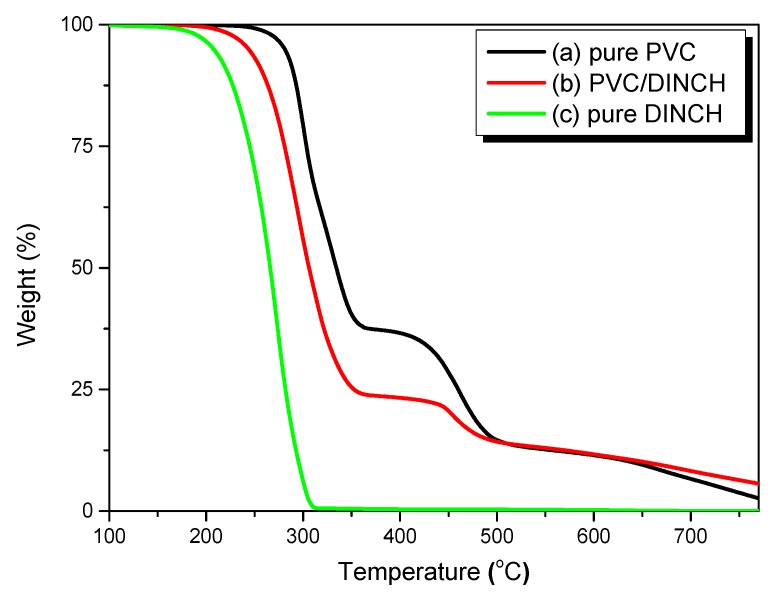
Thermogravimetric analyses (TGA) of (a) the pure PVC, (b) the PVC/DINCH = 100/60 blend, and (c) pure DINCH.

Because DINCH is among the most promising plasticizers used as a replacement for DEHP in PVC, we first investigated the thermal properties of PVC/DINCH blends through TGA analyses. Figure 1 displays the TGA traces of pure PVC, pure DINCH, and the PVC/DINCH = 100/60 blend, measured from 25 to 800 °C at a heating rate of 20 °C/min. Pure DINCH displayed its thermal decomposition at a temperature of *T*_d10_ = 213 °C, without providing a char yield, as displayed in Figure 1c. The pure PVC displayed two main thermal decomposition temperatures during the degradation process. The pyrolysis of PVC is complex when compared with those of other thermoplastic polymeric materials; it depends on the type and amount of plasticizer used [1,35]. The first thermal decomposition of PVC was initiated by the elimination of HCl at a relatively low temperature (300 °C and *T*_d10_ = 290 °C)—the so-called “dehydrochlorination” stage, during which polyene radicals and unsubstituted aromatics are released, as displayed in Figure S2a. Aromatic products and various degrees of char were formed during the second thermal decomposition stage at approximately 460 °C from DTG curves (Figure S2a) [35]. The solid residue of the char yield was approximately 2.7 wt% after completion of the PVC pyrolysis, corresponding to ash formation. After blending with DINCH, the value of *T*_d__10_ was 265 °C, because of the lower thermal decomposition temperature of pure DINCH. The char yield increased to 6 wt%, presumably because of ash formation from the reaction of DINCH with PVC. The lower thermal stability of the PVC/DINCH blend system, compared with that of pure PVC, encouraged us to add MA-POSS to potentially improve the thermal stability. Because of the possibility of intermolecular hydrogen bonding between PVC and the MA-POSS nanoparticles, we used DSC, TGA, DMA, and FTIR spectroscopy to perform initial investigations into the miscibility, thermal properties, and hydrogen bonding interactions of binary blends of PVC/MA-POSS.

### 3.2. Thermal Properties and FTIR Spectra of PVC/MA-POSS Blends

**Figure 2:**
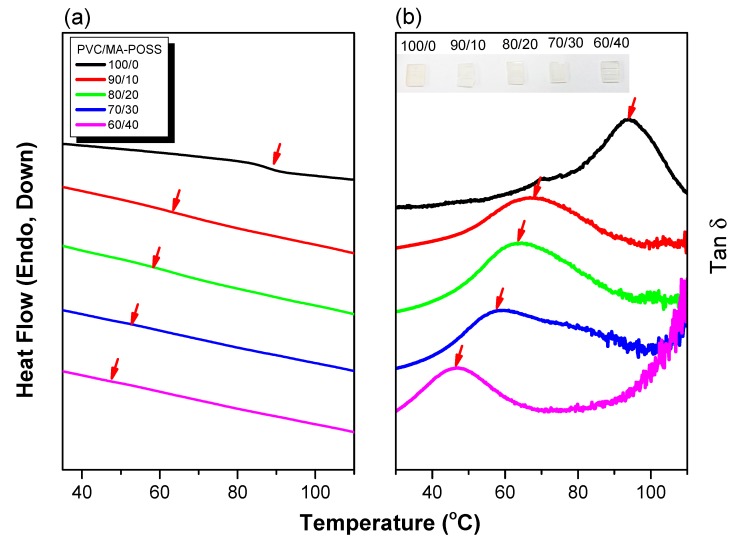
(**a**) Differential scanning calorimetry (DSC) and (**b**) dynamic mechanical analysis (DMA) of various PVC/MA-POSS blends; the inset to (**b**) displays the transparent films obtained after DMA analyses.

Figure 2 presents the results of DSC and DMA analyses of PVC/MA-POSS blends in the range 30–110 °C. The pure PVC exhibited a value of *T*_g_ of 89 °C, based on DSC analysis (Figure 2a), and 94 °C, based on DMA analysis (Figure 2b). It is not unusual for the value of *T*_g_ determined using DMA to be higher than that from DSC analysis, typically by 5–10 °C. In addition, pure MA-POSS has a reported glass transition temperature of −55 °C (Figure S1), and is a pale-yellow heavy oil [33,34].

**Figure 3:**
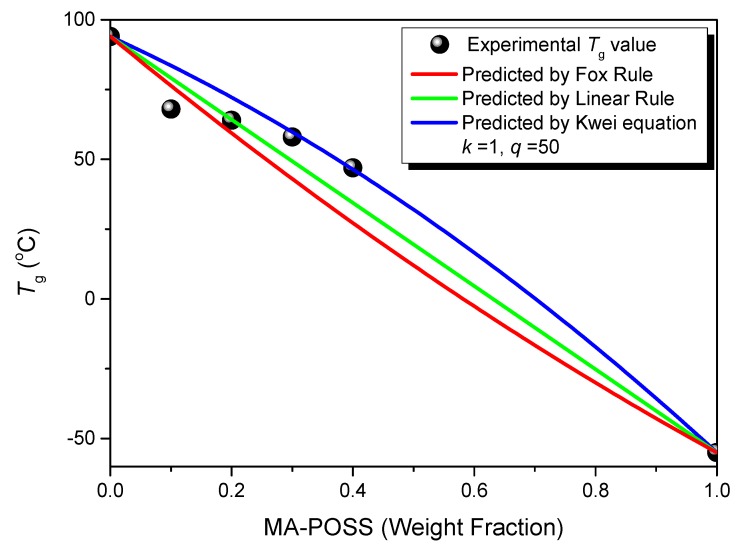
Experimental values of *T*_g_ of PVC/MA-POSS blends and those predicted by the Fox and linear rules and the Kwei equation.

The values of *T*_g_, determined using both DSC and DMA, decreased significantly upon increasing the MA-POSS concentration. When 10 wt% of MA-POSS was incorporated into the PVC matrix, the value of *T*_g_ decreased to 63 (DSC) and 68 (DMA) °C, approximately 26 °C lower than that of pure PVC, indicating that the plasticizing effect of MA-POSS towards PVC was significant. Further increasing the MA-POSS content to 20, 30, and 40 wt% caused the values of *T*_g_ to decrease to 64, 58, and 47 °C, respectively, based on DMA analysis, confirming that MA-POSS is a promising plasticizer for PVC, consistent with previous results [33,34]. In addition, all the PVC/MA-POSS blends possessed only a single glass transition temperature, implying that these blends were fully miscible. Furthermore, the values of *T*_g_ were between those of the two individual compounds, as expected. More importantly, all the PVC/MA-POSS blends were transparent and flexible, even after thermal treatment (DMA), as displayed in the inset to Figure 2b. Figure 3 presents the values of *T*_g_ of the PVC/MA-POSS blends, as determined using DMA. Although the glass transition temperatures of the PVC/MA-POSS blends were higher than the values predicted by the Fox rule and the linear rule, they were predicted using the Kwei equation [36]:

(1) $$T_{g}=\frac{W_{1}T_{g1}+kW_{2}T_{g2}}{W_{1}+kW_{2}}+qW_{1}W_{2}$$

where *W*_1_ and *W*_2_ are the weight fractions and *T*_g1_ and *T*_g2_ are the glass transition temperatures of the MA-POSS and the PVC, respectively, while *k* and *q* are fitting constants. We obtain values of *k* and *q* of 1 and 50, respectively, for the PVC/MA-POSS blends, indicating that specific intermolecular interactions were occurring between the PVC and MA-POSS nanoparticles.

**Figure 4:**
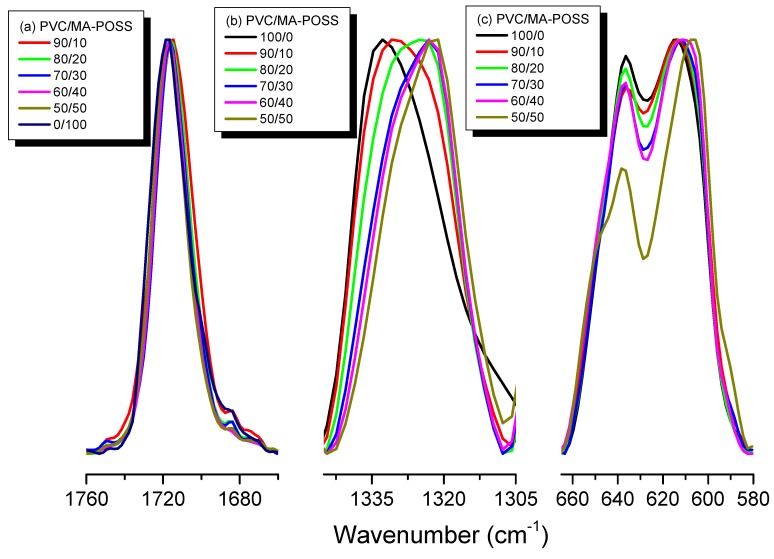
FTIR spectral analyses of the (**a**) C=O groups of MA-POSS, (**b**) C–H deformation of the CH_2_ units of the PVC, and (**c**) C–Cl stretching vibration of the PVC, for various PVC/MA-POSS blends recorded at room temperature.

FTIR spectroscopy can provide information about the noncovalent interactions of both polar and nonpolar groups through hydrogen bonding or dipole–dipole interactions [37,38,39,40]. Figure 4 presents the characteristic absorption signals for the (a) C=O units of MA-POSS, (b) C–H deformations of the CH_2_ units of PVC, and (c) C–Cl stretching vibrations of PVC in various PVC/MA-POSS blends. The free C=O groups of MA-POSS provided an absorption at 1717 cm^−1^; this signal shifted to 1714 cm^−1^ at 90 wt% of PVC concentration, as displayed in Figure 4a. In addition, the signal for the CH_2_ units of PVC shifted from 1332 to 1321 cm^−1^ (Figure 4b), while its C–Cl units shifted significantly from 614 to 606 cm^−1^ (Figure 4c), after blending with the MA-POSS nanoparticles. These results indicate that intermolecular hydrogen bonding existed between the H–CCl units of the PVC and the C=O groups of the MA-POSS, as displayed in Scheme 1d. Such weak hydrogen bonding interactions have also been observed in many other binary blend systems [41,42,43].

**Figure 5:**
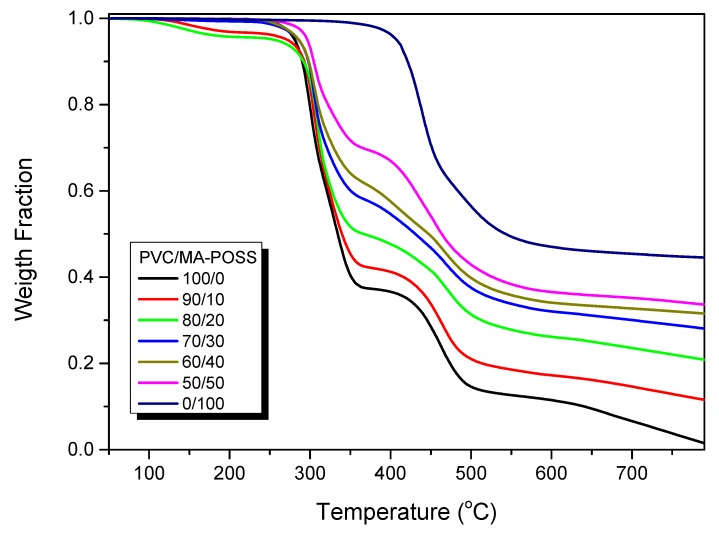
TGA analyses of PVC/MA-POSS blends of various compositions.

Figure 5 displays the TGA curves of various PVC/MA-POSS blends, recorded from 25 to 800 °C at a heating rate of 20 °C/min, and the corresponding DTG curves are displayed in Figure S3. The thermal stability of pure MA-POSS was greater than that of pure PVC, which possessed a value of *T*_d__10_ of 421 °C and a char yield of 44.5 wt%. Upon increasing the content of MA-POSS, the first thermal decomposition temperature and the char yield both increased. When the MA-POSS concentration reached 50 wt%, the value of *T*_d__10_ was close to 306 °C, approximately 16 °C higher than that of pure PVC, while the char yield also increased to 33.7 wt%, higher than the value [23.6 wt% = (44.5 + 2.7)/2] suggested by the simplified relationship of pure MA-POSS (44.5 wt%) + pure PVC (2.7 wt%)—again, probably because of ash formation from the reaction of MA-POSS with PVC. This result suggests that the rate of HCl emission during the first thermal decomposition procedure had decreased as a result of the presence of MA-POSS nanoparticles. The main thermal decomposition of MA-POSS overlapped with the second thermal decomposition process of PVC, making it difficult to identify, as displayed in Figure S3. In addition, the decomposition process of MA-POSS becomes obvious after increasing the MA-POSS concentration, becoming comparable with that of pure MA-POSS. The final step in the TGA curves corresponds to the pyrolysis of conjugated double bonds (e.g., of the polyenes formed in the first step of PVC decomposition), with the decomposition temperature and char yield both increasing upon increasing the MA-POSS concentration, because of the retarding effect of MA-POSS nanoparticles toward the possible residual ash [33,34]. Thus, our DSC, DMA, FTIR spectroscopic, and TGA analyses confirmed that the incorporation of MA-POSS nanoparticles could decrease the value of *T*_g_ of PVC, and increase both its thermal decomposition temperature and the char yield, while providing full miscibility with PVC, because of weak hydrogen bonding in the PVC/MA-POSS blends. As a result, MA-POSS has the potential to be a suitable replacement for traditional plasticizers, e.g., DEHP, and could be used as a blood bag, to avoid the DEHP coming into contact with tissues or body fluids.

### 3.3. Thermal and Mechanical Properties of PVC/DINCH/MA-POSS Ternary Blends

**Figure 6:**
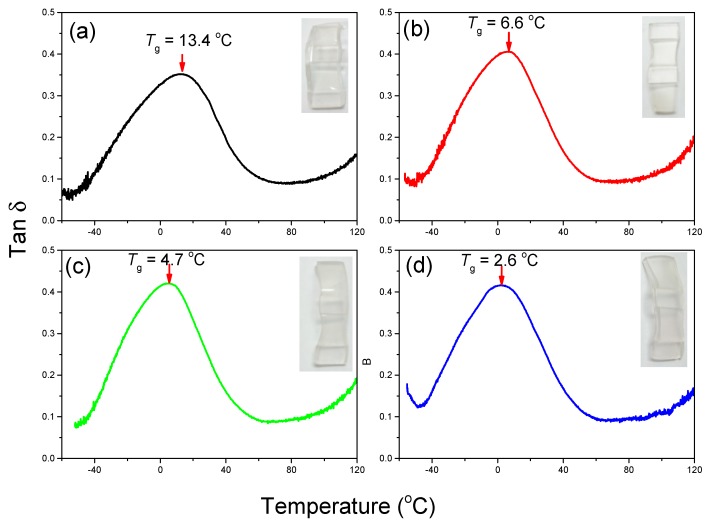
DMA analyses of various PVC/DINCH/MA-POSS blends: (**a**) 100/60/0, (**b**) 100/60/5, (**c**) 100/60/10, and (**d**) 100/60/15. The inset displays the transparent films obtained after DMA analysis of each composition.

To solve the problem of the lower thermal stability of PVC/DINCH blend systems compared to pure PVC, we tested the effect of adding MA-POSS nanoparticles. Figure 6 presents DMA analyses of PVC/DINCH/MA-POSS ternary blends at various MA-POSS contents. The value of *T*_g_ of the binary PVC/DINCH blend was 13.4 °C. Increasing the MA-POSS content to 5, 10, and 15 phr caused the value of *T*_g_ to decrease to 6.6, 4.7, and 2.6 °C, respectively, based on DMA analysis, suggesting that MA-POSS remains a promising plasticizer for PVC/DINCH blends. In addition, we observed transparent behavior for all the PVC/DINCH/MA-POSS ternary blends, with flexibility, even after thermal treatment through DMA analysis (see inset to Figure 6).

**Figure 7:**
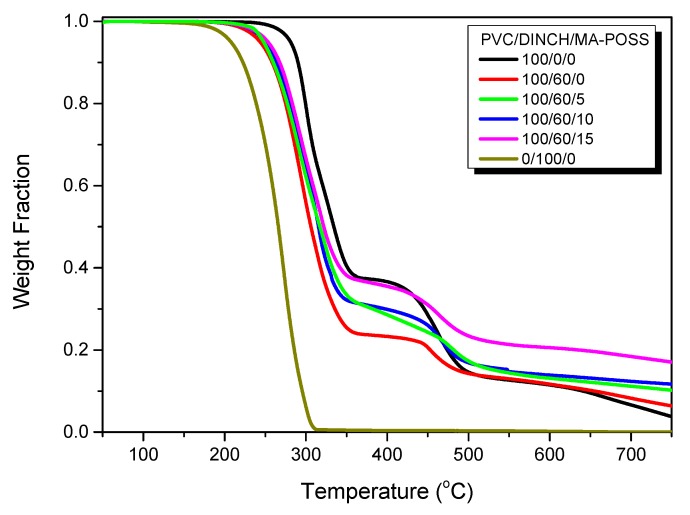
TGA analyses of PVC/DINCH/MA-POSS blends of various compositions.

Figure 7 presents the TGA curves of PVC/DINCH/MA-POSS ternary blends at various MA-POSS contents, measured from 25 to 800 °C at a heating rate of 20 °C/min. The corresponding DTG curves are also displayed in Figure S4. Increasing the MA-POSS content in the PVC/DINCH binary blend caused the first thermal decomposition temperature and the char yield to increase, as displayed in Figure S4. When the MA-POSS content reached 15 phr, the value of *T*_d_ was close to 271 °C, approximately 6 °C higher than that of the PVC/DINCH binary blend. Furthermore, the char yield also increased to 17.3 wt%, 11.3 wt% higher than that of the PVC/DINCH binary blend.

**Figure 8:**
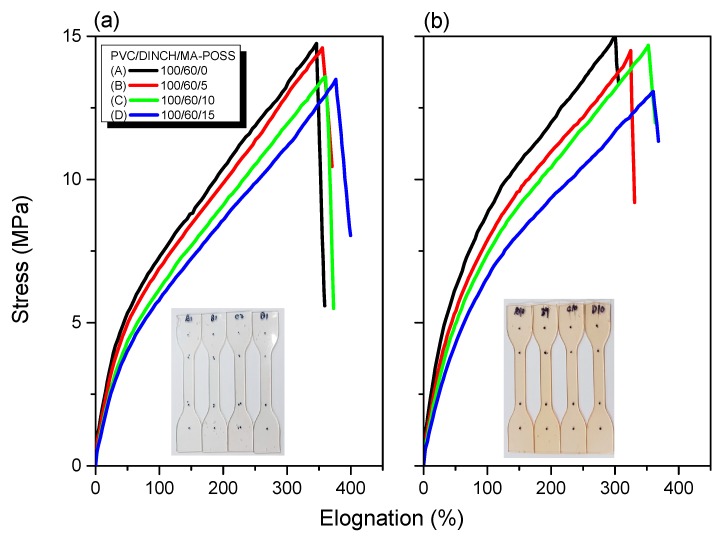
Stress–strain curves of PVC/DINCH/MA-POSS blends at various MA-POSS contents (**a**) before and (**b**) after thermal annealing at 100 °C for 7 days. The inset displays the tensile test samples used in this study (**a**) before and (**b**) after thermal annealing.

Figure 8 displays the results of tensile tests of PVC/DINCH/MA-POSS ternary blends at various MA-POSS contents (a) before and (b) after thermal annealing at 100 °C for 7 days. All the PVC/DINCH/MA-POSS blends exhibited high elongation behavior, even after thermal annealing, and we could briefly observe that the modulus and tensile strength decreased, and elongation increased, upon increasing the content of MA-POSS, both before and after thermal annealing.

**Figure 9:**
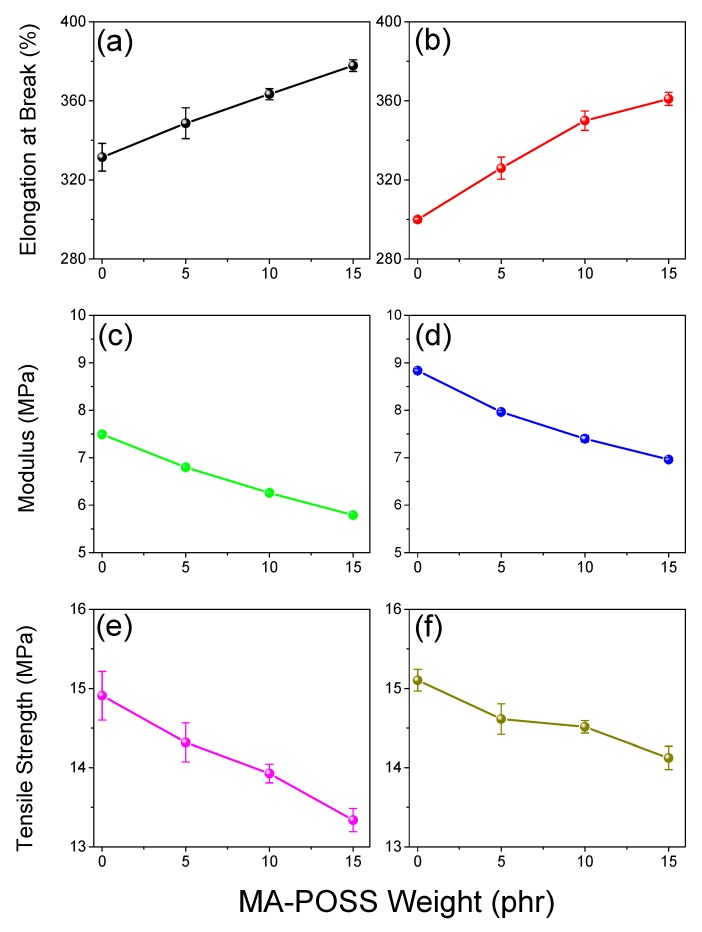
Mechanical properties of PVC/DINCH/MA-POSS blends at various MA-POSS contents (**a**,**c**,**e**) before and (**b**,**d**,**f**) after thermal annealing at 100 °C for 7 days: (**a**,**b**) elongation properties, (**b**,**d**) moduli, and (**c**,**f**) tensile strengths.

Figure 9 summarizes the mechanical properties. Elongation decreased, but the modulus and tensile strength increased, after thermal annealing. We also observed transparency for all the PVC/DINCH/MA-POSS ternary blends, as well as flexibility. They became pale yellow after thermal annealing at 100 °C for 7 days, as displayed in Figure 8.

## 4. Conclusions

We have examined the thermal properties, miscibility, hydrogen bonding interactions, and mechanical properties of PVC/MA-POSS binary blends and PVC/DINCH/MA-POSS ternary blends. Employing DSC, DMA, TGA, and tensile tests, we confirmed that the incorporation of MA-POSS nanoparticles into the blends occurred with full miscibility; they decreased the value of *T*_g_, the modulus, and tensile strength, but increased the thermal decomposition temperature, char yield, and elongation properties, because weak hydrogen bonding existed between the C=O groups of the MA-POSS and the H–CCl units of the PVC, as determined through FTIR spectral analyses. As a result, MA-POSS appears to be a suitable candidate for replacing traditional plasticizers, e.g., DEHP, in PVC matrices.

## Supplementary Materials

The following are available online at https://www.mdpi.com/2073-4360/11/10/1711/s1.
