# Supplementary material for: Using Methacryl-Polyhedral Oligomeric Silsesquioxane as the Thermal Stabilizer and Plasticizer in Poly(vinyl chloride) Nanocomposites
Source: Polymers (Basel). 2019 Oct 18;11(10):1711. doi: 10.3390/polym11101711 (PMC6835242; doi:10.3390/polym11101711)
Supplement: Supplementary file 1 [file polymers-11-01711-s001.pdf]

Supporting Information for

Using Methacryl-Polyhedral Oligomeric Silsesquioxane as the Thermal Stabilizer and Plasticizer in Poly(vinyl chloride) Nanocomposites

Yu-Kai Wang,[1] Fang-Chang Tsai[2,*], Chao-Chen Ma,[3] Min-Ling Wang,[3] and Shiao-Wei Kuo[1,4*]

[1]Department of Materials and Optoelectronic Science, Center of Crystal Research, National Sun Yat-Sen University, Kaohsiung 80424, Taiwan. E-mail: gba01123@gmail.com (Y.K W.)

[2]Hubei Key Laboratory of Polymer Materials, Key Laboratory for the Green Preparation and Application of Functional Materials (Ministry of Education), Hubei Collaborative Innovation Center for Advanced Organic Chemical Materials, School of Materials Science and Engineering, Hubei University, Wuhan 430062, China

[3]UPC Technology Corporation, Unnamed Road, Kaohsiung 832, Taiwan. E-mail: cj.maa@upc.com.tw (C. C. Ma) Vini.Wang@upc.com.tw, (M. L. W.)

[4]Department of Medicinal and Applied Chemistry, Kaohsiung Medical University, Kaohsiung 807, Taiwan

Correspondence: tfc0323@gmail.com (F.-C.T.); kuosw@faculty.nsysu.edu.tw (S.-W.K.); Tel.: +886-7-525-4099 (S.-W.K.); Tel.: +86-27-88661729 (F.-C.T.)

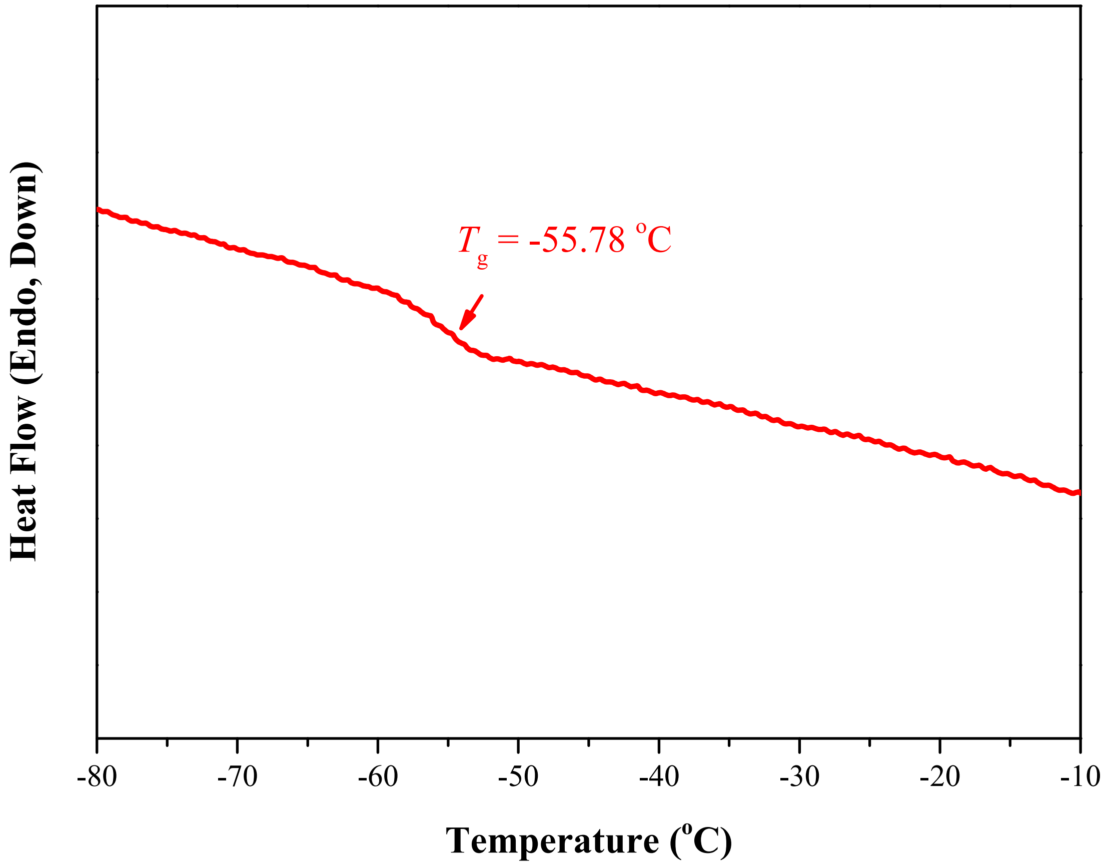

Figure S1: The Tg value of MA-POSS based on DSC analysis.

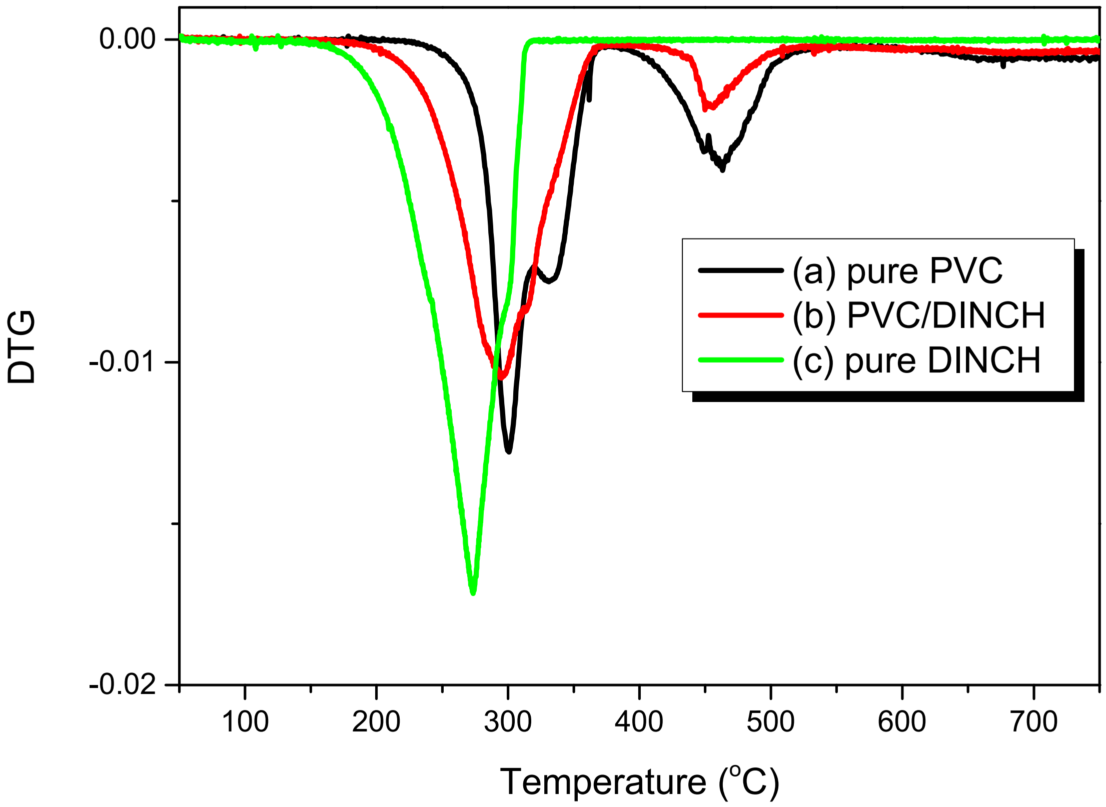

Figure S2: DTG curves of (a) the pure PVC, (b) the PVC/DINCH = 100/60 blend, and (c) pure DINCH.

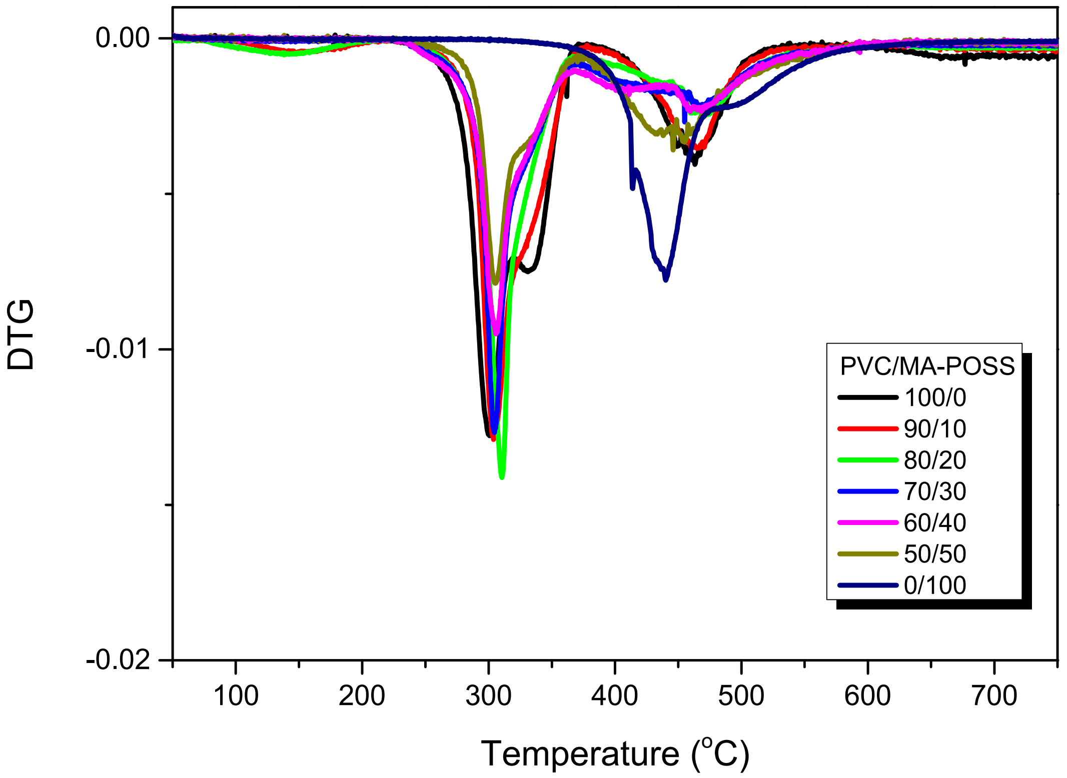

Figure S3: DTG curves of PVC/MA-POSS blends of various compositions.

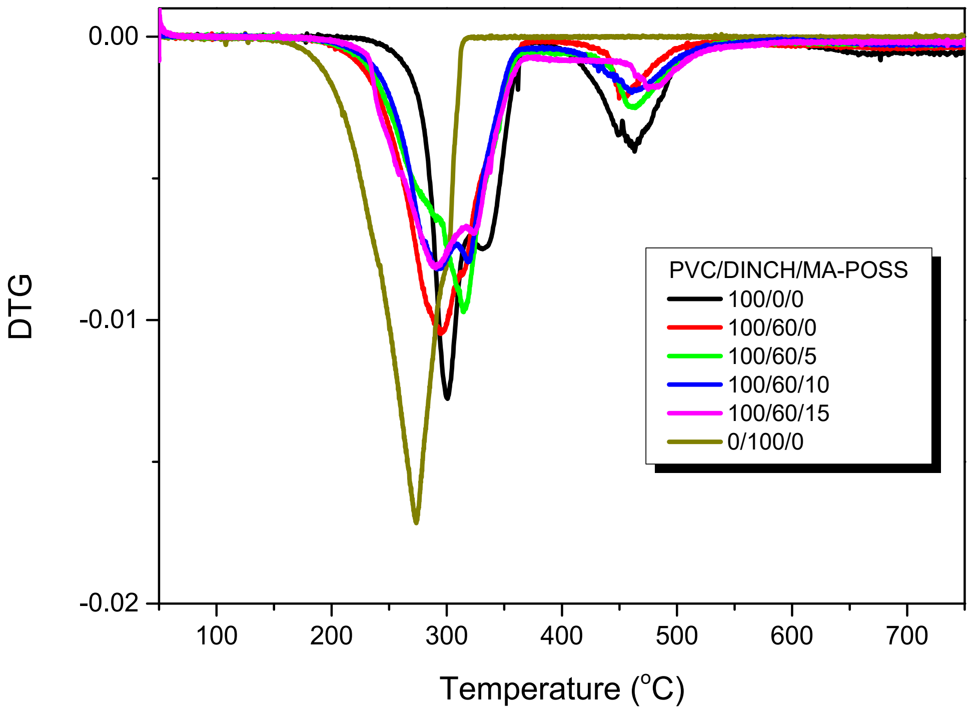

Figure S4: DTG curves of PVC/DINCH/MA-POSS blends of various compositions.
